# Effects of varying wheat levels on growth performance, intestinal barrier, and cecal microbiota of broilers

**DOI:** 10.3389/fvets.2024.1409125

**Published:** 2024-07-29

**Authors:** Leilei Wang, Bin Wei, Xuemeng Si, Yanqun Huang, Huaiyong Zhang, Wen Chen

**Affiliations:** ^1^Key Laboratory of Animal Biochemistry and Nutrition, Ministry of Agriculture, College of Animal Science and Technology, Henan Agricultural University, Zhengzhou, China; ^2^Laboratory for Animal Nutrition and Animal Product Quality, Department of Animal Sciences and Aquatic Ecology, Ghent University, Ghent, Belgium

**Keywords:** wheat, broiler, growth performance, intestinal integrity, cecum microbiota

## Abstract

**Introduction:**

The study aimed to investigate the potential effects of varying wheat levels in broiler diets on growth performance, intestinal barrier, and cecal microbiota.

**Methods:**

Day-old male broilers were fed the same diet until 10 d of age. Then they were randomly assigned to 1) the low-level wheat group, where inclusion of 15.0% and 25.0% wheat in the grower and finisher diet, respectively, 2) the medium-level wheat group with 30.0% and 40.0% of wheat in the grower and finisher periods; and 3) the high-level wheat dietary group, in which the grower and finisher diets contained 55.77% and 62.38% of wheat, respectively.

**Results:**

Dietary treatments unaffected the body weight at 39 d, whereas incorporating high wheat in diets significantly increased the feed intake and reduced the feed conversion ratio from 10 to 39 d (*p* < 0.05). Except for increased phosphorus digestibility in the high wheat group, dietary treatments had no significant effect on the apparent digestibility of dry matter, crude protein, and ether extract. Meanwhile, the broilers that consumed the medium and high content of wheat presented a higher villus height and the ratio of villus height to crypt depth than those fed the low-level wheat birds. Feeding the medium-level wheat enhanced ileal integrity and depressed the expression of proinflammatory cytokines in the ileum. The addition of high levels of wheat reduced the Chao1 index and the abundance of *Lactobacillaceae*, *Bacteroidaceae*, and *Ruminococcacea* in cecal content, which probably decreased the metabolism of histidine, sulfur-containing amino acids, and the biosynthesis of lysine.

**Discussion:**

These results support the medium-level wheat diet improved intestinal barrier function and had no deleterious effects on the growth performance of broiler; dietary inclusion of high wheat reduced the feed conversion rate, which might be associated with the disturbed gut microbiota and decreased metabolism of amino acids.

## 1 Introduction

The corn-based diet is by far the most used for intensively reared poultry on account of the palatability and high nutritional value of corn (1). In recent decades, because of the rapid development of poultry farming, the shortage of global supply, and the rising price demand to seek the replacement of corn in animal husbandry. The widespread use of wheat as energy feed for livestock in Europe sheds light on substituting corn with wheat. A study on broilers found that adding 40% wheat to the diet had no significant decrease in the body weight (BW) of broilers (2). The data from the growing-finishing pigs also assumed that the diet with 10–60% wheat did not alter the BW and weight gain of pigs (3). However, a compromised BW was noticed in the broilers fed the wheat-based diets when compared to those offered the corn-based diets (4). Moreover, it was pointed out that broilers fed a diet with 42 or 63% wheat possessed increased weight gain relative to the birds receiving corn-based diets (5, 6). These findings highlighted the significance of searching for an appropriate level of wheat in the diets of domestic birds.

From the perspective of nutrients, wheat contains lower starch than corn, but it has higher crude protein and amino acid levels, including lysine, tryptophan, and threonine (7). Of note, the contents of fiber and non-starch polysaccharides (NSP) in wheat are higher than in corn (8), which results in increased viscosity, thereby separating substrates from endogenous enzymes and decreasing nutrient utilization consequently (9). Indeed, the data from broilers who fed either wheat- or corn-soybean diets confirmed that the wheat-based increased approximately 3 times less viscosity of ileal chyme than the corn-based diet (10). The higher fiber could also encapsulate other nutrients to shield from digestive enzymes and thus might reduce their digestibility (11), evidenced by the poor digestibility of crude protein and ether extract in pigs fed the wheat-soybean diet as compared to those the corn-soybean diet (12, 13). Accordingly, the incorporation of exogenous in wheat-based diets has often been used to decrease digesta viscosity and improve utilization of nutrients in poultry (14). This reason why dietary β-glucanase and/or xylanase supplementation notably increased the nutrient utilization and growth performance in wheat-fed birds (4–6). The positive roles exerted by NSP-degrading enzymes in laying performance and feed conversion ratio were also observed in laying hens (15). Nevertheless, the employment of enzymes such as xylanase, cellulase, pectinase, etc., was noticed to fail to promote nutrient digestibility and productive performance in domestic birds (16). In addition to the sources and activities of enzymes, as well as the type of basal diet, the limited hydrolytic capacity of these enzymes may be due to the influent gut environment and the proteolytic stability of enzymes (17). Hence, rationalizing the wheat replacement for corn in the diets of broilers is one of the alternative ways to improve NSP utilization and growth performance.

In practice, the 15 and 25% wheat are commonly used in commercial feed formulations of broilers in grower and finisher diets, respectively. Considering the no obvious influence of 40% wheat (2) on BW and the positive roles of the diet with 42 or 63% wheat in weight gain of broilers (5, 6), the dose of 15–63% wheat designed to evaluate the effects of wheat as a replacement for corn on growth performance, intestinal barrier, and cecal microbiota of broilers in the iso-energy and -protein diet, as well as supplying the multienzyme complex. These outcomes might provide valuable insights into the optimal level of wheat substitution for corn in broiler diets, contributing to cost-effective production in the broiler industry and animal welfare.

## 2 Materials and methods

### 2.1 Animal, diets, and experimental procedures

All research procedures were proved by the animal care committee of Henan Agricultural University (No. HNND20190306). A total of 480 1-day-old male AA broilers were fed the same starter diets until 10 days, then they were allocated into one of three groups based on the BW, i.e., (1) the low-level wheat group, in which the wheat addition ratios were 15.0 and 25.0% on the grower and finisher periods, respectively; (2) the medium-level wheat group with the 30.0 and 40.0% of wheat in the grower and finisher diets, respectively; and (3) the high-level wheat group, where the grower and finisher diets contained 55.77 and 62.38% of wheat, respectively. Each group included 8 pens with 20 broilers per pen. The diet was formulated to meet the nutrient requirements of AA broilers (18) and were supplied as pellets (Table 1). In addition, the multienzyme complex was supplemented in all diets as shown in Table 2. Birds were raised in floor pens (1.0 m × 0.9 m) and allowed to freely access diets and water during the whole trial. The initial room temperature was set at approximately 34°C and subsequently reduced to 24°C by 20 days. The light program was 23 L:1 D during days 10–39.

**Table 1:** The ingredient and calculated composition of basal starter and finisher diets (dry matter basis).

Items	Starter diet (1–10 days)	Grower diet (11–21 days)			Finisher diet (22–39 days)		
		Low	Medium	High	Low	Medium	High
Ingredients, %							
Corn	35.18	35.18	22.27	0.00	32.34	19.28	0.00
Wheat	15.00	15.00	30.00	55.77	25.00	40.00	62.38
Flour	15.0	15.0	15.0	15.0	10.0	10.0	10.0
Soybean oil	1.0	1	1.1	1.3	3.9	4.1	4.2
Soybean meal (46%)	20.2	20.2	18	14.2	16	13.8	10.5
Peanut meal (50%)	3.0	3.0	3.0	3.0	3.0	3.0	3.0
Corn gluten meal (60%)	3.0	3.0	3.0	3.0	3.0	3.0	3.0
Meat meal	2.2	2.2	2.2	2.2	2.2	2.2	2.2
Sodium chloride;	0.20	0.20	0.20	0.20	0.25	0.25	0.25
Limestone	0.98	0.98	0.95	0.90	1.07	1.04	1.00
Montmorillonite	0.2	0.2	0.2	0.2	0	0	0
Dicalcium phosphate	1.60	1.60	1.57	1.55	0.8	0.8	0.77
Choline (60%)	0.10	0.10	0.10	0.10	0.08	0.08	0.08
L-lysine HCl (70%)	0.90	0.90	0.95	1.08	1.02	1.10	1.20
DL-Methionine (99%)	0.22	0.22	0.22	0.23	0.20	0.20	0.21
L-Threonine	0.25	0.25	0.27	0.30	0.27	0.28	0.34
Broiler complex enzyme	0.30	0.30	0.30	0.30	0.30	0.30	0.30
Sodium bicarbonate	0.1	0.1	0.1	0.1	0.1	0.1	0.1
Sodium butyrate (98%)	0.04	0.04	0.04	0.04	0.04	0.04	0.04
Premix^*^	0.53	0.53	0.53	0.53	0.53	00.53	0.53
Total	100.0	100.0	100	100.0	100.0	100.0	100.0
Calculated content, %							
AME, Kcal/Kg	2,950	2,950	2,950	2,950	3,150	3,150	3,150
Crud protein	21.5	21.5	21.5	21.5	20	20	20
Dry matter	86.82	86.82	87.23	87.94	87.16	87.57	88.18
Crud fiber	2.5	2.5	2.45	2.35	2.34	2.28	2.2
Calcium	0.88	0.88	0.88	0.88	0.75	0.75	0.75
Total phosphorus	0.67	0.67	0.68	0.68	0.59	0.59	0.6
Available phosphorus	0.45	0.45	0.45	0.45	0.38	0.38	0.38
Digestible Lysine	1.28	1.28	1.28	1.28	1.25	1.25	1.25

*Premix providing per kg of diet: vitamin A (retinyl acetate), 9,985 IU; vitamin D_3_ (cholecalciferol), 4,293 IU; vitamin E (dl-α-tocopherol acetate), 39.3 mg; vitamin K_3_ (menadione), 3.975 mg; vitamin B_1_ (thiamine), 4.293 mg; vitamin B_2_ (riboflavin), 9.67 mg; niacin, 62.96 mg; D-pantothenic acid, 21.75 mg; vitamin B_6_ (pyridoxine-HCl), 4.60 mg; vitamin B_12_ (cyanocobalamine), 0.038 mg; folic acid, 2.16 mg; biotin, 0.2 mg; Fe (FeSO_4_.H_2_O), 41.44 mg; Cu (CuSO_4_.5H_2_O), 10 mg; Zn (ZnO), 80.5 mg; Mn (MnO), 85.56; I (Ca(IO_3_)_2_), 0.992 mg; Se (Na_2_O_3_Se), 0.304 mg.

**Table 2:** The exogenous enzymes composition.

Item	Activity, IU/g	Proportion, %
Xylanase	100,000	2.00
Cellulase	8,000	0.75
β-glucanase	50,000	0.80
Mannase	50,000	0.30
Pectinase	30,000	0.50
α-galactosidase	2,000	0.50
Others	–	95.15
Total		100.00

### 2.2 Data collection and sampling

The broilers were weighed at d 10, 14, 21, 28, 35, and 39 of age, and the feed intake (FI) from 10 to 39 d was recorded on a replicate basis. The weight gain and feed conversion rate indicated as the ratio of feed consumption to weight gain (F: G) were calculated from 10 to 39 days. The number of death and culling birds was registered to determine the survival proportion. The cost of producing meat, which is expressed as Yuan/kg meat, was calculated based on the price of raw ingredients and the feed conversion rate. Feed and fecal samples were collected and stored at −80°C for the test of the apparent digestibility of nutrients. At 39 days, one bird was selected according to the average BW of each pen for sampling. Blood collection from the jugular vein and separating serum. Subsequently, these birds were euthanasia, the gizzard, spleen, thymus, and bursa of Fabricius were weighed for calculating the relative weight. The duodenum, jejunum, ileum, and cecum were dissected, and length and weight were obtained after removing the thyme. Segments of 1 cm length from the middle of the duodenum, ileum, jejunum, and cecum were dissected and immersed in phosphate-buffered formaldehyde for histology analysis. The mid-ileum (removing 1 cm right in the middle for histology analysis) was collected for gathering the mucosa. Cecal contents were obtained for microbiota analysis.

### 2.3 Apparent digestibility of nutrients

The collected feed and fecal samples were dried in a constant-temperature oven at 65°C before being crushed through a 40-mesh sieve. The apparent digestibility of the nutrients was evaluated using acid-insoluble ash (AIA) as an endogenous indicator. The ether extract of both the diet and the fecal samples was extracted using the Soxhlet method, while the crude protein was determined using the Kjeldahl nitrogen determination method. Calcium and phosphorus were determined by ethylene diamine tetraacetic acid titration and ammonium metavanadate colorimetry, respectively.

### 2.4 Intestinal histological analysis

The formaldehyde-fixed intestinal samples were dehydrated, embedded, and sliced into 5-μm thick sections. These sections were stained with hematoxylin–eosin (H&E). At least 10 well-oriented villi units were used for the measurement of muscular thickness, villus height, and crypt depth. The ratio of villus height to crypt depth was then calculated.

### 2.5 Ileum gene expression

Total RNA was extracted from the ileal mucosa, and the quantity and quality of RNA were assessed using spectrometry and denaturing agarose gel electrophoresis, respectively. The cDNA was then synthesized using a cDNA reverse transcription kit (Takara, Dalian, China). The obtained cDNA was used for gene expression analysis using SYBR green qPCR master mix (Takara). The amplification conditions consisted of an initial denaturation step at 95°C for 15 s, followed by 40 cycles of amplification at 95°C for 30 s and 60°C for 34 s, with a final melting curve analysis. Primers were designed and listed in Table 3. *β-actin* was used as the housekeeping gene for normalizing the expressions of the target genes.

**Table 3:** The primers for real-time-PCR.

Gene	Gene ID	Primer sequences (5′→3′)	Product length, bp
*ZO-1*	XM_046925214.1	F: GAAGAGAGCACAGAACGCAG	123
		R: CACTTGTGGCAAGCTGAAGT	
*Claudin-1*	NM_001013611.2	F: TCTGGTGTTAACGGGTGTGA	117
		R: GTCTTTGGTGGCGTGATCTT	
*Occludin*	NM_205128.1	F: CGTTCTTCACCCACTCCTCC	107
		R: CCAGAAGACGCGCAGTAAGA	
*CDH1*	NM_001039258.3	F: AGCCAAGGGCCTGGATTATG	157
		R: GATAGGGGGCACGAAGACAG	
*IL-6*	NM_204628.1	F: CCCTCACGGTCTTCTCCATA	100
		R: CTCCTCGCCAATCTGAAGTC	
*IL-1β*	NM_204524.1	F: GTTTTTGAGCCCGTCACCT	117
		R: CACGAAGCACTTCTGGTTGA	
*TNF-α*	NM_204267.1	F: ACTGGGCGGTCATAGAACAG	120
		R: AGATGGGAAGGGAATGAACC	
*β-actin*	NM_205518.1	F: GTCCACCGCAAATGCTTCTAA	78
		R: TGCGCATTTATGGGTTTTGTT	

ZO-1, zonula occludens-1; CDH1, cadherin 1. IL-6, Interleukin-6; TNF-α, Tumor necrosis factor alpha.

### 2.6 16S rDNA amplicon sequencing of cecal microbiota

The total DNA in cecal content was extracted using a DNA stool mini kit (Qiagen, Valencia, United States). Subsequently, these DNA was subjected to the assessment of the integrity. Primers 515 F (5′-GTGYCAGCMGCCGCGGTAA-3′) and 806 R (5′-GGACTACHVGGGTWTCTAAT-3′) were used to amplify the hypervariable V3-V4 regions of the 16S rDNA gene. Then, the resulting PCR products were sequenced on an Illumina PE250 platform (BGI, Shenzhen, China). The obtained sequences were processed using FLASH (v1.2.11) and USEARCH (v7.0.1090) for alignment and clustering. All effective reads were clustered into operational taxonomic units (OTUs) with a similarity threshold of 97%. The representative sequence of each OTU was aligned against the Greengene database for taxonomy analysis. The alpha diversity was estimated with the Chao 1, ACE, Simpson, and Shannon indexes, and related function enrichment was performed using the Kyoto Encyclopedia of Genes and Genomes (KEGG) analysis.

### 2.7 Serum inflammatory and immunoglobulin

The concentrations of interleukin (IL)-1, IL-6, IL-10, tumor necrosis factor-alpha (TNF-α), and transforming growth factor beta (TGF-β) were determined using commercial kits following the manufacturer’s instructions. Serum immunoglobulin (Ig), including IgG, IgA, and IgM, were analyzed using commercial chicken-specific enzyme-linked immunosorbent assay (ELISA) kits. All kits were purchased from Nanjing Jiancheng Bioengineering Institute (Nanjing, China).

### 2.8 Statistical analysis

Data were analyzed using the JMP software (SAS Institute). The correlation coefficients between microbiota and growth performance were calculated by simple regression analysis. After checking the normal distribution and homogeneity of variance, the significance was detected via a one-way repeated measure analysis of variance (ANOVA) with Tukey’s *post hoc* comparison. A significant value was set at *p* < 0.05. Results were expressed as means ± standard deviation.

To define the relationship between the BW and age, von Bertalanffy, logistic, and Gompertz models were selected to assess the coefficient of determination (R^2^) by the Gauss-Newton algorithm. The logistic model was finally selected as the optimized model for BW as follows:


$$BW=\frac{A}{\left(1-B*\exp^{\left(-K*\mathrm{day}\right)}\right)}$$

In which, A is the asymptotic BW, B is a constant of integration without biological interpretation, and K is the maturity rate. The age of maximal growth rate is on (lnB)/K age with A/2 BW.

## 3 Results

### 3.1 Growth performance

**Figure 1:**
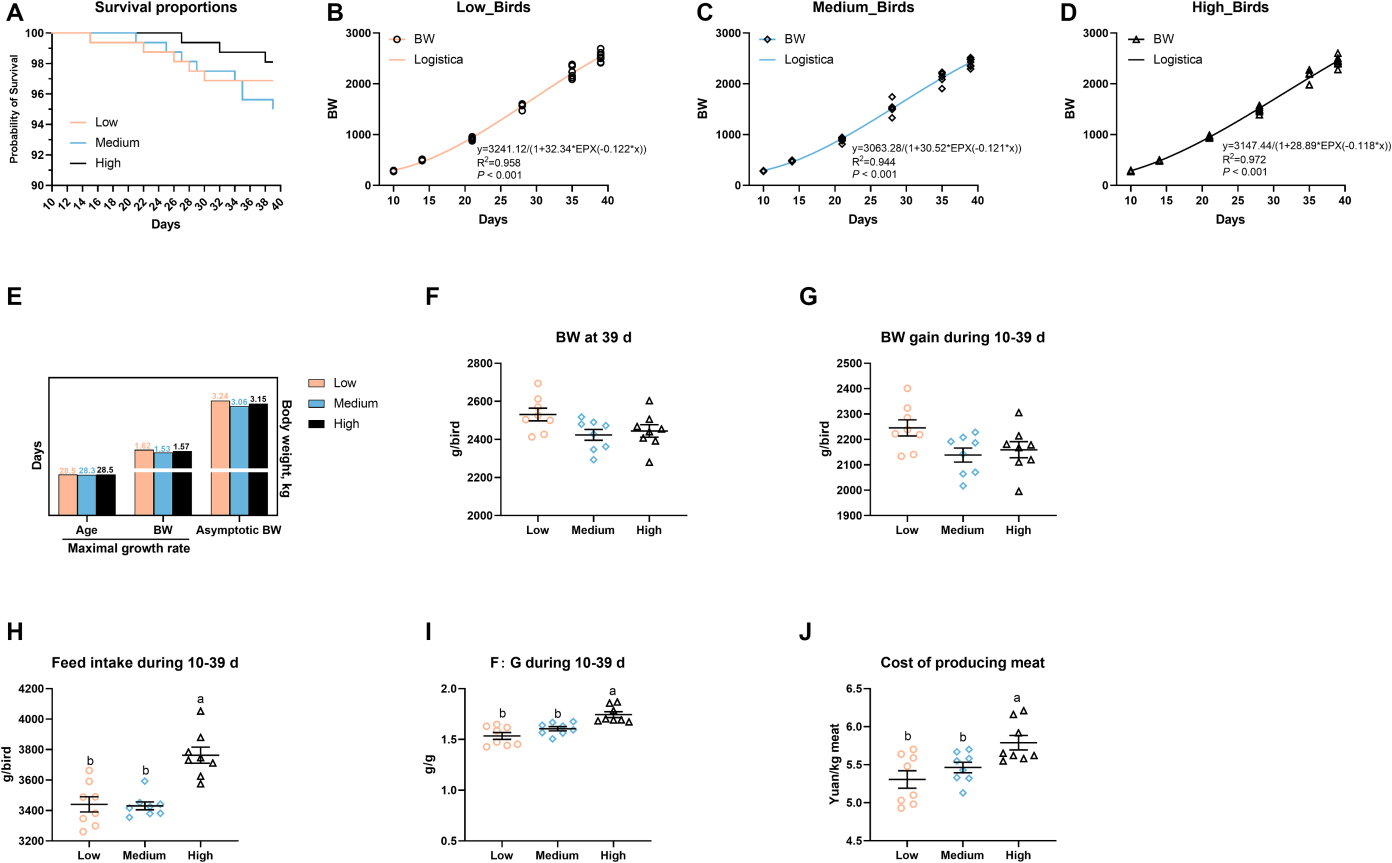
Effects of the partial replacement of corn with wheat on the growth performance of broilers. **(A)** Survival from 10 to 39 days. **(B–D)** Body weight (BW) of birds on different days and modeling on the growth curve in response to the replacement of corn with the logistic model, and **(E)** the maximal growth rate and asymptotic BW were calculated. **(F)** BW at 39 days. **(G)** Weight gain, **(H)** feed intake, **(I)** the ratio of feed intake to gain (F: G), **(J)** cost of producing meat during 10–39 days. Values are means and standard deviation (*n*  =  8). ^a,b^Mean values with different letters are significantly different (*p* < 0.05).

As shown in Figure 1, there was no obvious difference in terms of survival proportions among the three groups (Figure 1A). Using the logistic model, the medium- and high-level wheat addition failed to affect the age and BW of maximal growth rate, whereas the increased wheat proportion decreased the asymptotic BW of broiler chickens, i.e., 3.24, 3.06, and 3.15 kg in the low-, medium-, high-level wheat groups, respectively (Figures 1B–E). With the increasing wheat levels in diets, both the weight gain from 10 to 39 days and BW at 39 days were decreased (*p* > 0.05; Figures 1F,G). Of note, the incorporation of high-level wheat in diets significantly increased the FI during 1–39 days (*p* < 0.05), and thus resulted in higher F: G and the cost of producing meat relative to the low- and medium-level wheat groups (Figures 1H–J).

### 3.2 Apparent digestibility

Dietary treatments had no significant effect on the apparent digestibility of dry matter, crude protein, ether extract, and calcium (*p* > 0.05) (Table 4). However, as compared with the low-level wheat group, the dietary addition of high-level wheat notably increased the apparent digestibility of phosphorus (*p* < 0.05).

**Table 4:** Effects of the partial replacement of corn with wheat on the apparent digestibility of broilers on day 39.

Item	Low	Medium	High	*p*-value
Dry matter, %	81.10 ± 4.48	82.24 ± 1.09	82.69 ± 2.52	0.754
Crud protein, %	61.62 ± 6.28	71.55 ± 2.29	69.02 ± 8.70	0.124
Ether extract, %	93.13 ± 0.60	93.76 ± 1.00	93.63 ± 2.04	0.793
Calcium, %	65.53 ± 7.11	62.27 ± 4.63	61.38 ± 3.27	0.525
Phosphorus, %	56.41 ± 7.71^b^	63.25 ± 0.70^ab^	66.94 ± 3.48^a^	0.039

All the results were shown as mean ± standard deviation. Means without common superscript are significantly different at *P* < 0.05 (*n* = 4).

### 3.3 Gizzard and intestinal development

**Figure 2:**
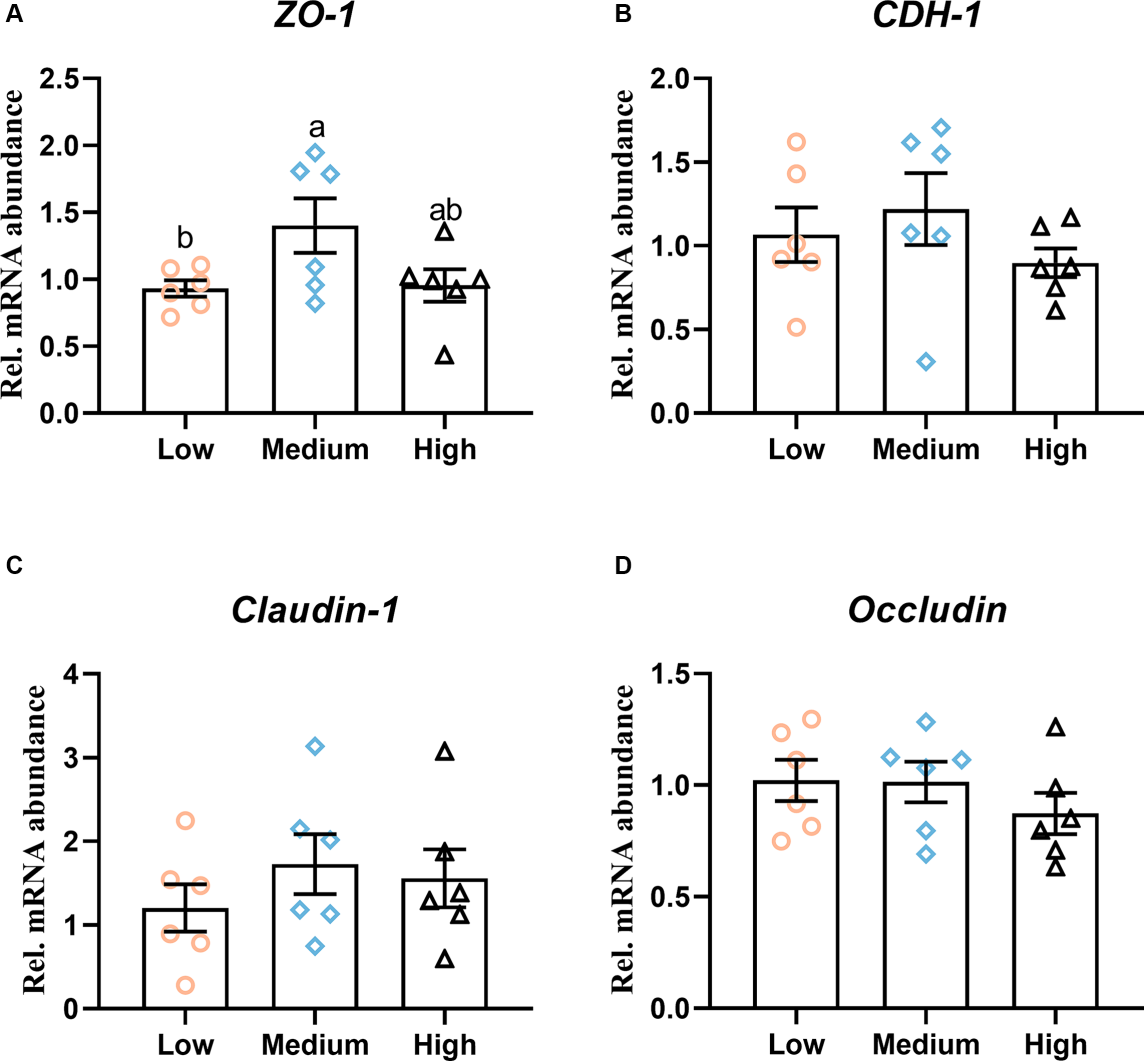
Intestinal integrity in response to dietary wheat supplementation. The mRNA level of **(A)** Zonula occludens-1 (*ZO-1*), **(B)** Cadherin 1 (*CDH1*), **(C)** *Claudin-1*, and **(D)** *Occiudin* in ileal mucosa. Values are means and standard deviation (*n* = 6). ^a,b^Mean values with different letters are significantly different (*p* < 0.05).

The effect of dietary treatments on digestive organ development is presented in Table 5. The inclusion of wheat in the diets had less influence on the weight of the glandular stomach, gizzard, small intestine, and cecum. Meanwhile, the dietary treatment of wheat did not change the absolute and relative length of the duodenum, jejunum, ileum, and cecum (*p* > 0.05). Regarding the effect of dietary treatments on intestinal morphology, the trial treatment had notable effects on intestinal morphology, villus height, crypt depth, and their ratio of duodenum. Compared to the low- and medium-level wheat group, the high-level wheat supplementation significantly decreased the crypt depth of jejunum (*p* < 0.05). Meanwhile, the broilers that consumed the medium and high content of wheat took out a higher villus height and the ratio of villus height to crypt depth than those fed the low-level wheat group in the ileum (*p* < 0.05) (Table 5). In addition, the outcomes of RT-qPCR showed that the medium level of wheat addition notably upregulated the mRNA expression levels of zonula occludens-1 (*ZO-1*) as compared to the low wheat diet (Figure 2A). No significant difference was observed regarding the transcription of cadherin 1 (*CDH1*), *claudin-1*, and *occludin* among the three groups (Figures 2B–D).

**Table 5:** Effects of the partial replacement of corn with wheat on the intestinal development of 39-d-old broilers.

Item	Low	Medium	High	*P*-value
Relative weight, g/100 g body weight				
Glandular stomach	0.54 ± 0.15	0.52 ± 0.10	0.43 ± 0.10	0.184
Gizzard	0.86 ± 0.28	0.83 ± 0.18	0.68 ± 0.11	0.183
Duodenum	0.62 ± 0.11	0.57 ± 0.09	0.56 ± 0.10	0.445
Jejunum	0.92 ± 0.10	1.06 ± 0.20	1.27 ± 0.83	0.390
Ileum	0.91 ± 0.27	0.74 ± 0.12	0.90 ± 0.16	0.170
Cecum	0.62 ± 0.11	0.63 ± 0.14	0.67 ± 0.19	0.748
Relative length, cm/100 g body weight				
Duodenum	1.05 ± 0.09	1.07 ± 0.07	1.08 ± 0.12	0.775
Jejunum	2.09 ± 0.24	2.13 ± 0.33	2.38 ± 0.38	0.170
Ileum	2.19 ± 0.39	2.19 ± 0.58	2.21 ± 0.44	0.997
Cecum	0.64 ± 0.06	0.63 ± 0.09	0.59 ± 0.10	0.425
Intestinal morphology				
Duodenum				
Muscular thickness, μm	148.79 ± 24.31	165.17 ± 35.38	171.38 ± 52.12	0.500
Villus height, μm	1776.30 ± 197.80	1885.34 ± 183.42	2093.35 ± 461.93	0.138
Crypt depth, μm	216.98 ± 42.41	234.15 ± 31.51	224.44 ± 43.81	0.690
Villus height to crypt depth	8.36 ± 1.18	8.21 ± 1.61	9.35 ± 1.26	0.216
Jejunum				
Muscular thickness, μm	169.36 ± 56.37	181.65 ± 20.67	149.45 ± 24.60	0.245
Villus height, μm	1675.74 ± 299.15	1711.70 ± 194.51	1785.43 ± 209.25	0.561
Crypt depth, μm	214.48 ± 34.51^a^	231.45 ± 64.03^a^	169.01 ± 18.76^b^	0.025
Villus height to crypt depth	8.10 ± 2.49	8.12 ± 3.24	10.68 ± 1.82	0.094
Ileum				
Muscular thickness, μm	179.94 ± 34.66	204.90 ± 46.31	221.15 ± 57.66	0.236
Villus height, μm	828.66 ± 53.40^b^	1282.27 ± 93.68^a^	1340.90 ± 72.15^a^	<0.001
Crypt depth, μm	179.03 ± 13.26	175.38 ± 38.75	163.56 ± 39.52	0.623
Villus height to crypt depth	4.64 ± 0.37^b^	7.69 ± 2.05^a^	8.54 ± 1.75^a^	0.002

All the results were shown as mean ± standard deviation. Means without common superscript are significantly different at *P* < 0.05 (*n* = 8).

### 3.4 Cecal microbiome

**Figure 3:**
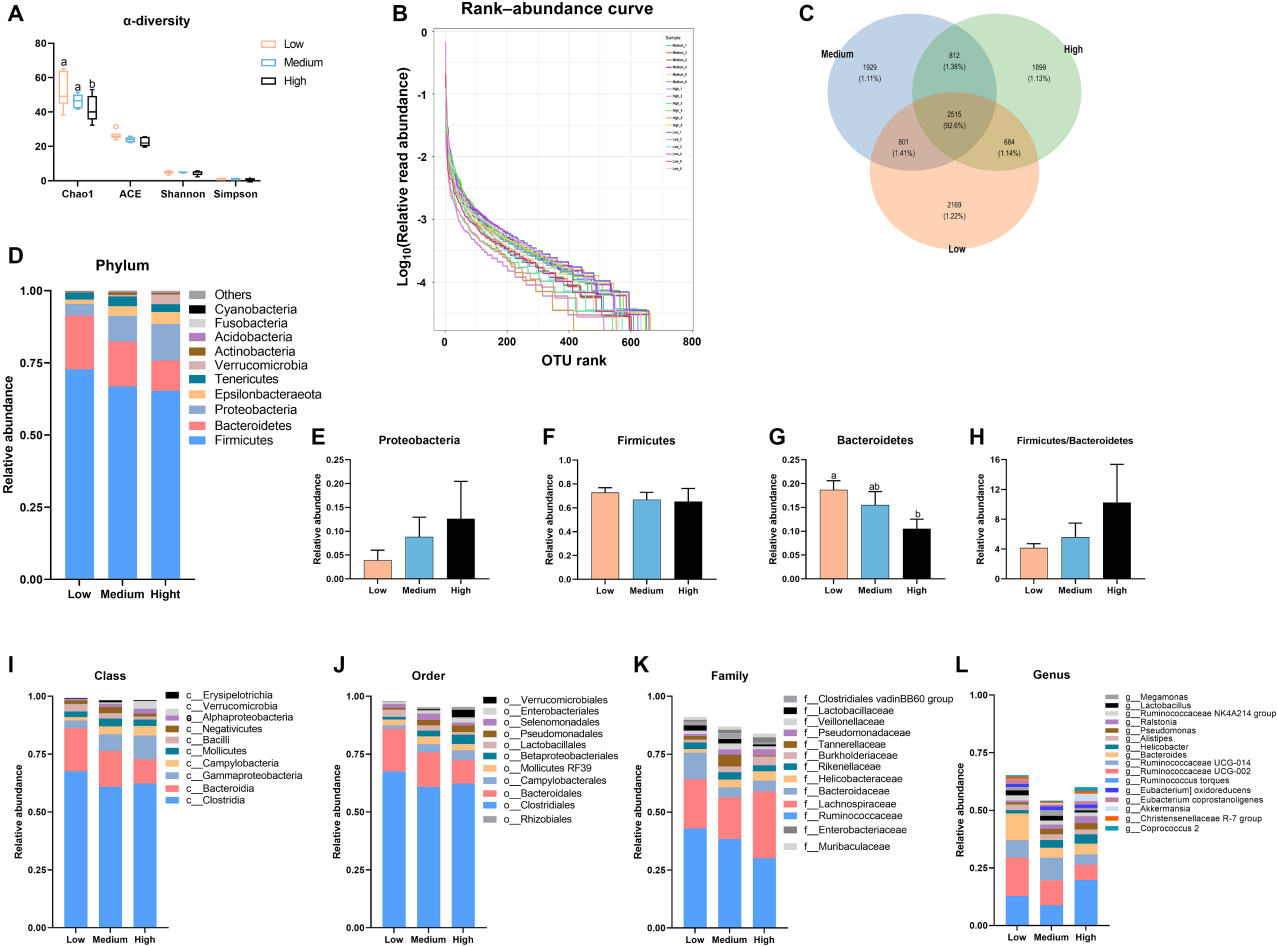
The alternation in the cecal microbiota of broilers fed the diets contained partial replacement of corn with wheat. **(A)** Alpha diversity was evaluated via Chao1, ACE, Simpson, and Shannon index. **(B)** Rank-abundance curve. **(C)** Venn diagram. **(D)** The distribution of the microbiota with the high abundance at the phylum level. **(E–H)** The proportion of Firmicutes, Bacteroidetes, and Proteobacteria, as well as the ratio of Firmicutes to Bacteroidetes. **(I–L)** The distribution of the microbiota with the high abundance at the class, order, family, and genus level. Values are means and standard deviation (*n* = 8). ^a,b^Mean values with different letters are significantly different (*p* < 0.05).

Originally, 669,181 raw reads were obtained. The raw sequences were quality trimmed and filtered, error models were constructed, amplicon sequence variants and operational taxonomic units (ASVs) were inferred, and forward and reverse reads were merged, and chimeras were removed following default settings or adjusted. It resulted in 606,932 reads (29,250–37,661 per sample) for cecal content. Subsequent bioinformatics was run and shown in Figure 3. When compared to the low-level wheat diet, the high-level wheat group remarkably reduced the Chao1 index (*p* < 0.05), but it did not change the ACE, Shannon, and Simpson indexes (Figure 3A). Figure 3B shows the trends in species abundance and homogeneity, in which the high wheat group exhibited a smaller horizontal range than the low- and medium-level wheat group, indicating lower abundance. According to the Venn diagram, the low, medium, and high wheat replacement for corn possessed 1.22, 1.11, and 1.13% specific flora in cecal microbiota, respectively (Figure 3C). Cluster analysis revealed that Firmicutes, Bacteroidetes, and Proteobacteria were the dominant bacterial communities in the cecal content of broilers (Figure 3D). At the phylum level, dietary wheat supplementation had little effect on the proportion of Proteobacteria and Firmicutes (Figures 3E,F). The high wheat inclusion significantly decreased the abundance of Bacteroidetes as compared to the low wheat groups, whereas it did not obviously change the ratio of Firmicutes and Bacteroidetes in cecal thyme (Figures 3G,H). With the addition of wheat, the abundances of *Bacteroidales*, *Bacilli*, and *Clostridia* were decreased, accompanied by an increase in the levels of *Campylobacteria* and *Gammaproteobacteria* at class level (Figure 3I). Reflecting the order level, an apparent decrease in the abundances of *Clostridiales*, *Bacteroidales*, and *Lactobacilales* was observed in the high-wheat group when compared to the low-wheat group, whereas the levels of *Betaproteobacteriale* and *Campylobacterales* were elevated by the high level of wheat supplementation (Figure 3J). Moreover, the high wheat supplementation also decreased the account of *Lactobacillaceae*, *Bacteroidaceae*, and *Ruminococcacea* relative to the low-wheat group at the family level (Figure 3K). At the genus level, the high-level wheat diet decreased the abundance of *Ruminococcin*, *Bacteroidetes*, and *Lactobacillus*, whereas it increased the proportion of *Helicobacter* and *Ralstonia* as compared to the low wheat diets group (Figure 3L).

**Figure 4:**
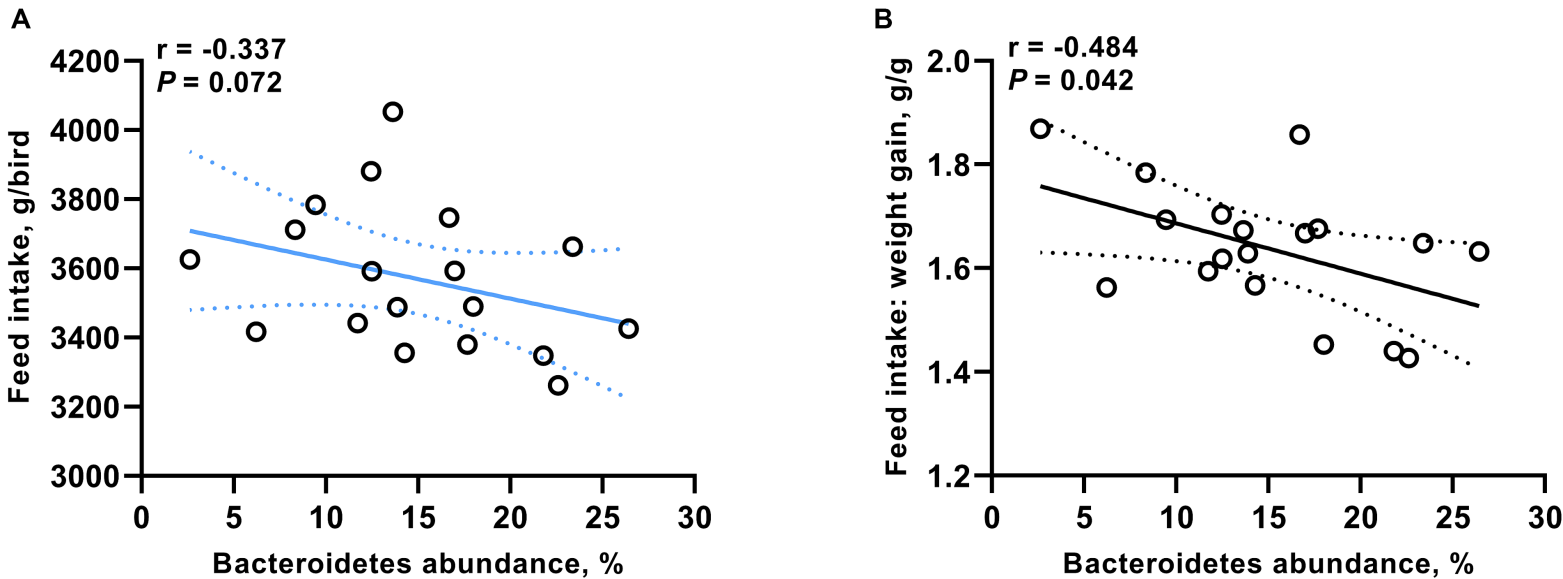
Correlation between the proportion of Bacteroidetes and both **(A)** feed intake and **(B)** the ratio of feed consumption to weight gain. Significance was accepted at *p* < 0.05.

We identified a significant correlation between Bacteroidetes (at the phylum level) and the growth performance of broiler chickens. As shown in Figure 4, the proportion of Bacteroidetes was negatively correlated with both FI (*r* = −0.337, *p* = 0.072) and the ratio of feed consumption to weight gain (*r* = −0.484, *p* = 0.042).

**Figure 5:**
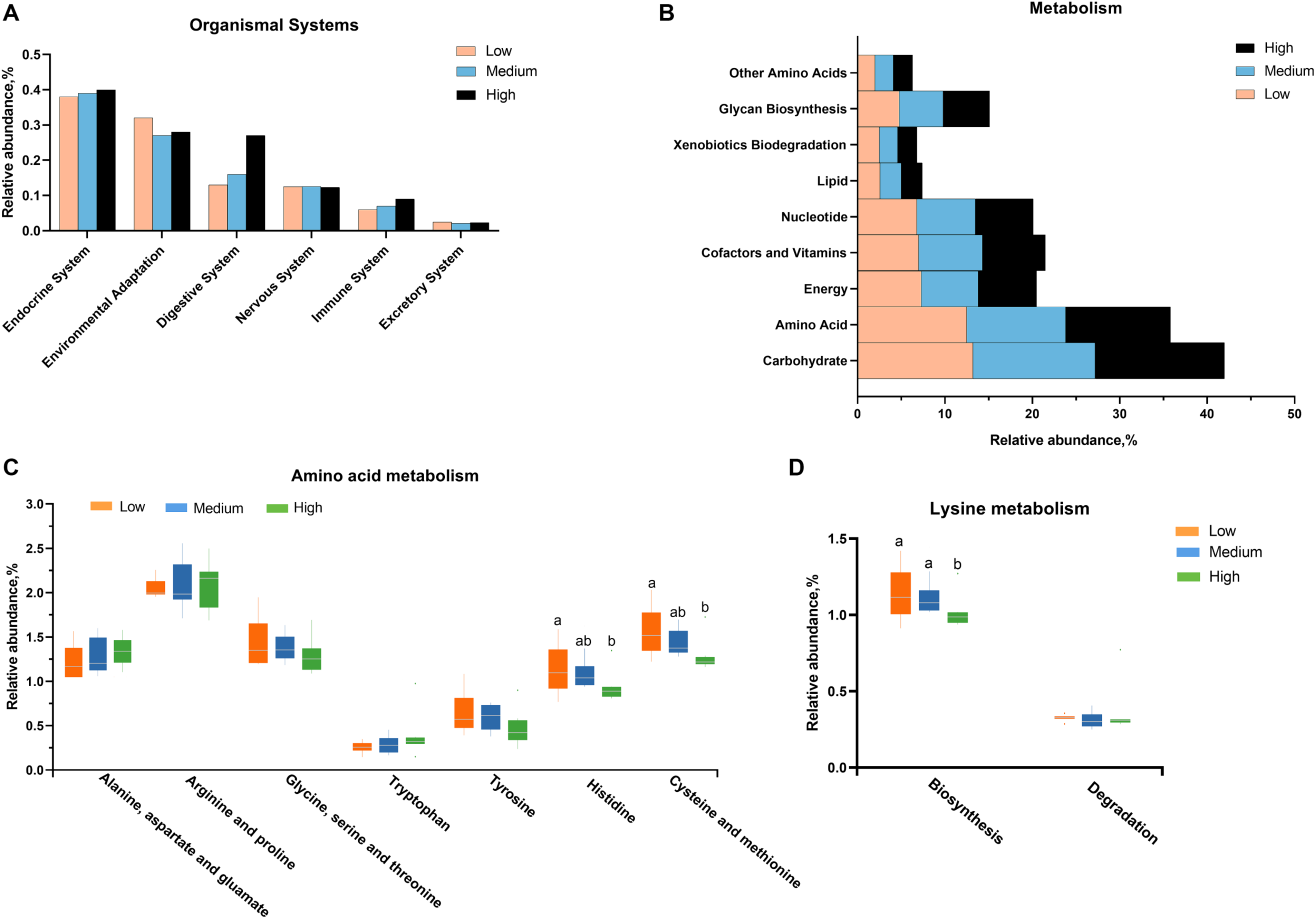
The related function enrichment using the Kyoto Encyclopedia of Genes and Genomes (KEGG) analysis according to the changed microbiota in **(A)** organismal system and **(B)** metabolism level, especially **(C,D)** amino acid metabolism. ^a,b^Mean values with different letters are significantly different (*p* < 0.05).

Furthermore, the predicted KEGG metabolic pathway suggests that the alternations in cecal microbiota might be associated with the environmental adaptation and digestive system (Figure 5A). At the metabolism level, the main influence of microorganisms is linked to the metabolism of carbohydrates and amino acids (Figure 5B). In detail, the supplementation of wheat did not significantly affect the metabolism of alanine, aspartate, glutamate, arginine, proline, glycine, serine, threonine, tyrosine, and tryptophan, but it decreased the histidine and sulfur-containing amino acids, as well as the biosynthesis of lysine (Figures 5C,D).

### 3.5 Inflammatory status and immune organ index

**Figure 6:**
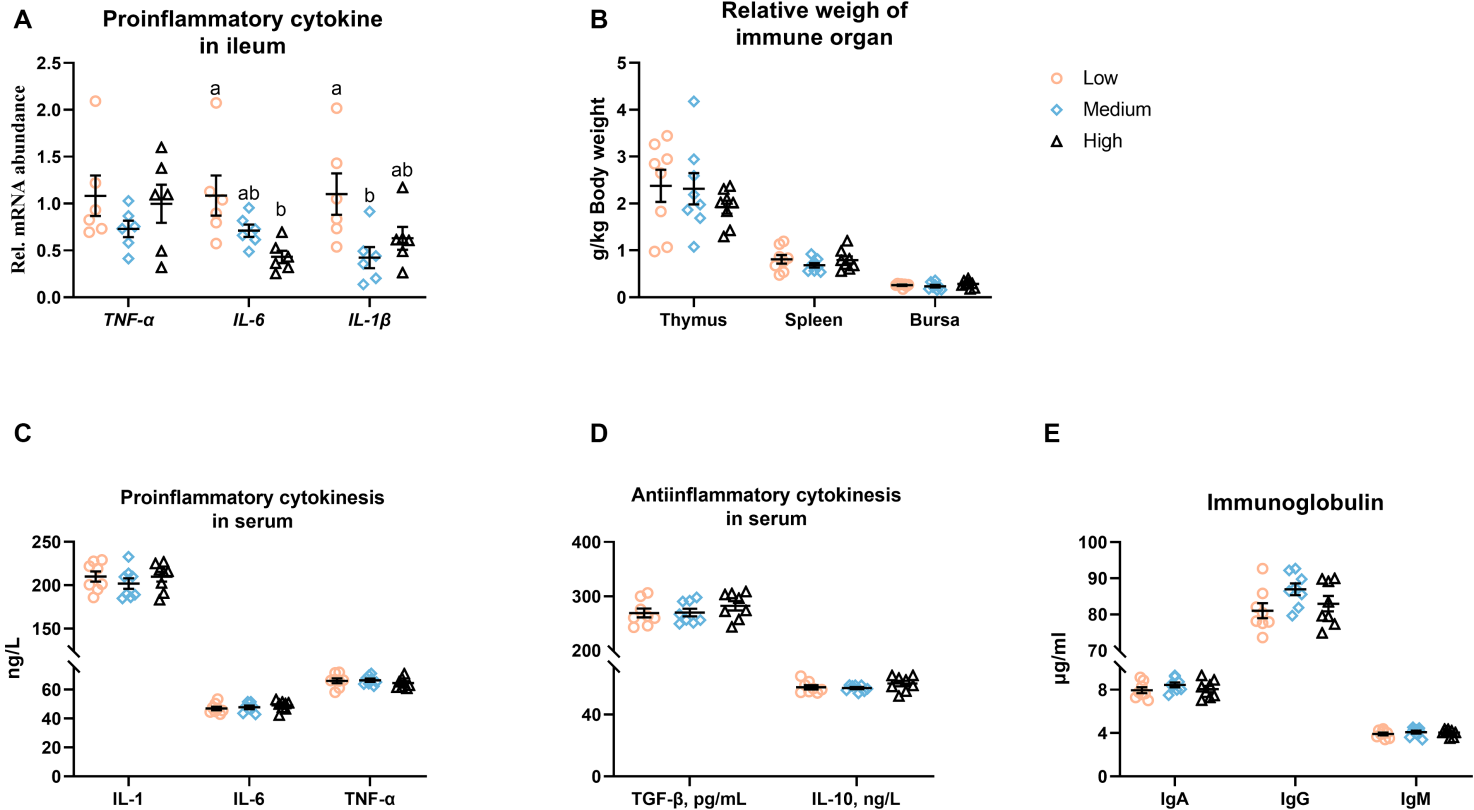
Influence of partial replacement of corn with wheat on **(A)** the mRNA abundances of pro-inflammatory cytokines in the ileum, **(B)** the relative weight of the immune organ, **(C–E)** the content of pro- and anti-inflammatory cytokines, as well as immunoglobulin (Ig) in serum. Values are means and standard deviation (*n* = 6–8). ^a,b^Mean values with different letters are significantly different (*p* < 0.05). IL, Interleukin; TNF-α, tumor necrosis factor alpha; TGF-β, transforming growth factor beta.

Figure 6 presents that the dietary inclusion was unaffected the mRNA level of *TNF-α*, while the medium- or high-level groups significantly downregulated (*p* < 0.05) the transcription of *IL-1β* and *IL-6*, respectively (Figure 6A). There was no obvious difference (*p* > 0.05) among the low-, medium-, and high-level wheat groups regarding the relative weight of immune organs, the content of serum inflammatory factors, and the immunoglobulin levels in serum (Figures 6B–E).

## 4 Discussion

Corn serving as the energy resource is the primary ingredient in poultry diets, and its shortage and rising price highlight the requirements for substitutes (19, 20). Multiple studies have provided stronger evidence that wheat might be an excellent option to replace corn in poultry diets (21–23). Considering the higher NSPs in wheat than corn, an anti-nutritive factor, the dose of wheat used in broiler diets is very important for optimizing the growth performance, intestinal homeostasis, and health status of birds. In this study, dietary inclusion of 30 and 40% wheat in the grower and finisher diets of broilers, respectively, had no deleterious effects on the growth performance, nutrient digestibility, and gut microbiota, whereas it improved the intestinal development and inflammatory status of broilers. When the supplemented levels of wheat increased to 55.77 and 62.38% in the grower and finisher diets, respectively, resulted in increased feed consumption, decreased feed conversion rate, and disturbed gut microflora.

The data from broilers confirmed that the wheat-based increased approximately 3 times more viscous of ileal digesta than the corn-based diet (10). Whereas the response of BW or weight gain to the wheat diet varies in pigs and birds. It was reported that broilers fed wheat diets possessed higher weight gain relative to the birds receiving corn-based diets (5, 6). Munyaka et al. assumed that it might be attributed to the corporation of insoluble NSP in ingredients or the higher weight mainly derived from the visceral organs (6). In addition, the inclusion of 40% wheat in a diet had no apparent effects on the BW of broilers (2). Feeding contained 10–60% of wheat in a corn-soybean meal-based diet supported that the supplementation of wheat did not significantly change the BW and weight gain in growing-finishing pigs (3). In the present study, the increased wheat levels in the diet led to declined BW, weight gain, and asymptotic weight of broilers according to the logistics analysis. A compromised BW was also observed in the broilers fed the wheat-based diets when compared to those offered the corn-based diets (4). For feed consumption and feed conversion rate, the delayed transit time and gut motility due to higher viscous, combined with the quarantine of substrates from endogenous enzymes, hindering FI is warranted in higher NSP-contained diets including the wheat-based diet (9), which was verified by the current study saying that the supplementation of the high-level wheat diet increased the FI and the F/G ratio from 10 to 39 days in broilers, resulting in higher the cost of producing meat. No significant difference was also noticed in broilers and pigs fed either wheat- or corn-soybean diets (3, 24). Mavromichalis et al. explained that the indistinctive FI in pigs might be a result of the high palatability of wheat to pigs (25). In our study, in accordance with previous research on birds (5), the increased feed consumption in high-level wheat diets suggested that the content of insoluble NSP in the diet of meat-type birds might be relevant, particularly in younger broilers.

Digestibility is defined as the proportion of food nutrients that are absorbed after ingestion, which is closely related to the fiber content and digesta passage rate (11). In addition to being resistant to endogenous enzymes, higher dietary fiber could encapsulate other nutrients and thus might reduce their digestibility (11). Therefore, the higher crude fiber and total NSP contained in wheat was pointed to result in the poor digestibility of crude protein and ether extract in pigs (12). Moreover, a high fiber concentration usually elevates the viscosity of the chyme and affects the passage rate, which might explain why the wheat-based diet exhibited a low digestibility of crude protein (13). On the contrary, a higher ileal digestibility of crude protein was observed in wheat-type diets than that of corn in pigs (12). Data from cattle also found a progressive improvement in the apparent digestibility of dry matter and crude protein with the increasing content of wheat in the diet (26). These discrepancies among studies probably were explained by the feed materials selected and the different fermentation capacities of trial objects. In the present study, dietary treatment did not notably alter the apparent digestibility of dry matter, crude protein, ether extract, and calcium of 39-day-old broilers. Consistent with the current outcomes, dietary inclusion of different levels of wheat unaffected the nutrient digestibility in growing-finishing pigs, including dry matter, crude fat, and crude protein (3). In general, the content of nutrients in a diet is closely linked to their digestibility (27). The comparable digestibility in this study might be attributed to the supplementation of the complex enzymes, especially xylanase and β-glucanase. In this regard, the positive roles of dietary β-glucanase and/or xylanase in nutrient utilization dependent on diet type manner have been multiply proven in birds (4–6). Of note, the release from phytate phosphorus that is rich in wheat due to the utilization of complex enzymes in this study might contribute to the higher digestibility of phosphorus in the high-level wheat diet. This was lined with previous documents assuming that wheat possessed a relatively higher digestibility of phosphorous relative to corn in pigs (28).

The development of the gastrointestinal tract plays a vital role in the nutrient utilization of livestock and poultry. A whole wheat diet was found to promote the enlargement and development of gizzard in broilers (29), which could promote the grinding of feed and improve the bonding of nutrients and enzymes (30). According to the published documents, the wheat diet increased the weight of the cecum, and the length of both the duodenum and ileum as compared to the corn diet (31). However, the varying levels of wheat supplementation failed to change the weight and the length of the intestine. In addition, measurement of intestinal morphology is conducive to assessing the capability of absorption of the gut to nutrients, and longer villi and/or shorter crypts may be linked to lower tissue turnover and excellent absorption function (32). When compared to the corn-based diets, the feeding of wheat-based diets was shown to reduce the villus height and the ratio of villus to crypt size, but it increased the crypt depth in the jejunum of laying hens (7). The wheat-based diet was found to induce a decreased villus width in broilers (2). Nevertheless, a study on growing pigs suggested that the addition of a wheat diet notably increased the villus height and the villus height/crypt depth ratio in the duodenum relative to the corn diet (24). Like these findings, the outcomes of the H&E staining in this study indicated that the high-level wheat diet reduced the crypt depth of the jejunum, and the medium- and high-level wheat diets improved the villus height and its ratio to crypt depth in the ileum. These beneficial effects of the wheat diet on intestinal morphology in this study probably derive from the supplementation of the complex enzyme, because the incorporation of an NSP-degrading enzyme in wheat-based diets remarkably augmented the villus height and reduced the crypt depth of duodenum in broilers (33). The data also implied that increased the length of the jejunum and the ileal absorptive surface area were not enough to reverse the decrease in BW caused by the high-level wheat in broilers.

Within the alternations of intestinal morphology, the medium-level wheat diet was noticed to enhance the ileal barrier function, evidenced by significantly upregulating the mRNA levels of *ZO-1*. Thus, the increased expression of tight junction proteins can be interpreted as a positive response for strengthening intestinal barrier function and integrity (34), which could improve intestinal resistance to pathogenic bacteria and depress inflammation outbursts (35). This might be explained by the downregulated expressions of pro-inflammatory cytokines, such as TNF-α, IL-6, and IL-1β, in the medium-level wheat group. However, the serum comparable concentrations of pro-inflammatory cytokines and anti-inflammatory factors including TGF-β and IL-10, as well as immunoglobulins indicated that the inhibited effects of the inclusion of medium-level wheat did not extend to systemic inflammation. Neither, in agreement with the previous data in broilers (2), the diet type did not alter the relative weight of immune organs, including the thymus, bursa, and spleen, in the current study.

The composition of the cecal microbiota is closely associated with the digestion and absorption of nutrients, immune status, and intestinal barrier functions of animals, and it is also influenced by the diet type (6, 24). For instance, the crude fiber contained in wheat could be directly fermented by microbiota as an energy material, which further affects the abundance of species of gut microbiota (36). A study on broilers found that the supplementation of wheat-based diets increased the account of *Escherichia coli* in the ileal thyme (2) and decreased the proportion of *Ruminococcus* in the caeca microbiota profile (10), compared to corn-based diets. It was also reported that the feeding of wheat-based diets improved the microbiota profile of cecal content in growing pigs as compared to the corn group, instructed by the decreased levels of *Escherichia-Shigella*, a well-known harmful bacterium, and increased the proportion of beneficial bacteria such as *Bifidobacterium* and *Lactobacillus* at the genus level (24). The discrepancy among these researchers may imply that the alterations of microbial composition rely on the dietary components, trial objects, environment, etc. In the present experiment, through 16 s sequencing for cecal microbiota, it was shown that the high substitution of corn with wheat reduced the Chao 1 index, implying that the high-level wheat inclusion decreased the microbial diversity in cecum content. At the phylum level, the abundance of Bacteroidetes was decreased with the supplementation of wheat in broilers. With the addition of wheat, the known beneficial bacterium abundances of *Lactobacillaceae*, *Bacteroidaceae*, and *Ruminococcacea* were decreased at the family level, as well as the proportion of *Ruminococcin*, *Bacteroidetes*, and *Lactobacillus* at the genus level. These data suggest that the high wheat supplementation as feedstuffs may decrease the colonization of profitable bacteria in cecal content, which might be linked with the alteration of growth performance, showed by a significantly negative correlated between the proportion of Bacteroidetes and both FI and the ratio of feed consumption to weight gain. Furthermore, the outcomes of KEGG analysis indicated the alterations in cecal microbiota were tightly associated with the change of organismal systems, especially the digestive system. In this process, the carbohydrates and amino acids were greatly enriched in metabolism levels. Specifically, the influence of microbiota had a slight effect on the metabolism of most amino acids, but it clearly decreased the histidine and sulfur-containing amino acids, as well as the biosynthesis of lysine. This was consistent with the findings of Ghazaghi et al., who observed the average of the standardized ileal digestibility (SID) of lysine, methionine, threonine, arginine, and histidine was reduced in quail chicks fed the wheat diet relative to the corn-based diets (37). A study on growing pigs pointed out that the supplementation of a wheat diet decreased the AID of histidine, leucine, threonine, alanine, and aspartate, but it increased the AID of lysine, phenylalanine, cysteine, and glutamate (12). This literature illustrates that the interaction between microbiota and amino acids probably implicates the nutrient utilization and growth of boilers in the current study.

There are some limitations in this study. The first is the setup of wheat dose, a successive level was required for determining the appropriate dose of wheat. The second is the limitation of parameters. The NSP in diet and viscosity in intestinal content was mentioned to significantly nutrient digestibility (13) and microbiota composition (36), whereas their contents are lacking in this study. In addition, the short-chain fatty acids (SCFAs) produced by microbial fermentation were also proved to play a critical role in intestinal development, but the data on SCFA levels were not shown in this study. The third is the evaluation of amino acids. The results of the KEGG analysis implied the deleterious effects of the high wheat diet might be related to the metabolism of amino acids, which needs to be confirmed in the future. Consequently, the inclusion of this study may overestimate or underestimate the effects of varying wheat levels in the diets of broilers on the growth performance, intestinal barrier, and cecal microbiota.

## 5 Conclusion

In conclusion, under the condition of supplementing exogenous enzymes, the diet with medium-level wheat (30% for the grower diet and 40% for the finisher diet, respectively) improved ileal morphology and integrity, thereby reducing intestinal inflammation, but it did not notably the growth performance of broiler chickens, whereas when the levels of wheat extended to 55.77 and 62.38% in grower and finisher diets, respectively, the high-level wheat diet significantly increased the feed consumption and decreased the feed conversion rate, which might be associated with the distribution of gut microbiota such as the decreased abundance of *Ruminococcin*, *Bacteroidetes*, and *Lactobacillus* at the genus level. Moreover, a negative correlation between the proportion of Bacteroidetes and both IF and the ratio of feed consumption to weight gain was also identified in this study.

## Data availability statement

The original contributions presented in the study are publicly available. This data can be found here: NCBI BioProject, PRJNA113735.

## Ethics statement

The animal study was approved by the Institutional Animal Care and Use Committee at the Henan Agricultural University. The study was conducted in accordance with the local legislation and institutional requirements.

## Author contributions

LW: Writing – original draft, Resources, Methodology, Investigation, Data curation, Conceptualization. BW: Writing – original draft, Visualization, Resources, Methodology, Investigation, Data curation. XS: Writing – review & editing, Resources, Methodology, Formal analysis, Data curation. YH: Writing – review & editing, Resources, Formal analysis, Data curation. HZ: Writing – original draft, Visualization, Supervision, Project administration, Funding acquisition, Data curation, Conceptualization. WC: Writing – review & editing, Supervision, Project administration, Funding acquisition, Formal analysis, Conceptualization.

## References

[1] Slominski BA. Recent advances in research on enzymes for poultry diets. Poult Sci. (2011) 90:2013–23. doi: 10.3382/ps.2011-01372, PMID: 21844268

[2] Ghiasvand AR Khatibjoo A Mohammadi Y Akbari Gharaei M Shirzadi H. Effect of fennel essential oil on performance, serum biochemistry, immunity, ileum morphology and microbial population, and meat quality of broiler chickens fed corn or wheat-based diet. Br Poult Sci. (2021) 62:562–72. doi: 10.1080/00071668.2021.1883551 33530744

[3] Han TH Hong JS Fang LH Do SH Kim BO Kim YY. Effects of wheat supplementation levels on growth performance, blood profiles, nutrient digestibility, and pork quality in growing-finishing pigs. Asian Australas J Anim Sci. (2017) 30:1150–9. doi: 10.5713/ajas.16.0838, PMID: 28183169 PMC5494489

[4] McCafferty KW Bedford MR Kerr BJ Dozier WA. Effects of age and supplemental xylanase in corn- and wheat-based diets on cecal volatile fatty acid concentrations of broilers1. Poult Sci. (2019) 98:4787–800. doi: 10.3382/ps/pez194, PMID: 31065717

[5] Kiarie E Romero LF Ravindran V. Growth performance, nutrient utilization, and digesta characteristics in broiler chickens fed corn or wheat diets without or with supplemental xylanase. Poult Sci. (2014) 93:1186–96. doi: 10.3382/ps.2013-03715 24795311

[6] Munyaka PM Nandha NK Kiarie E Nyachoti CM Khafipour E. Impact of combined beta-glucanase and xylanase enzymes on growth performance, nutrients utilization and gut microbiota in broiler chickens fed corn or wheat-based diets. Poult Sci. (2016) 95:528–40. doi: 10.3382/ps/pev333 26574039

[7] Abbasi Arabshahi H Ghasemi HA Hajkhodadadi I Khaltabadi Farahani AH. Effects of multicarbohydrase and butyrate glycerides on productive performance, nutrient digestibility, gut morphology, and ileal microbiota in late-phase laying hens fed corn- or wheat-based diets. Poult Sci. (2021) 100:101066. doi: 10.1016/j.psj.2021.101066, PMID: 33744611 PMC8010519

[8] Jaworski NW Lærke HN Bach Knudsen KE Stein HH. Carbohydrate composition and in vitro digestibility of dry matter and nonstarch polysaccharides in corn, sorghum, and wheat and coproducts from these grains. J Anim Sci. (2015) 93:1103–13. doi: 10.2527/jas.2014-8147, PMID: 26020887

[9] Bedford MR Schulze H. Exogenous enzymes for pigs and poultry. Nutr Res Rev. (1998) 11:91–114. doi: 10.1079/NRR19980007 19087461

[10] Nguyen HT Bedford MR Wu SB Morgan NK. Soluble non-starch polysaccharide modulates broiler gastrointestinal tract environment. Poult Sci. (2021) 100:101183. doi: 10.1016/j.psj.2021.101183, PMID: 34198096 PMC8253900

[11] Chen L Gao LX Huang QH Zhong RQ Zhang LL Tang XF. Viscous and fermentable nonstarch polysaccharides affect intestinal nutrient and energy flow and hindgut fermentation in growing pigs. J Anim Sci. (2017) 95:5054–63. doi: 10.2527/jas2017.1662, PMID: 29293707 PMC6292254

[12] Zhang S Zhong R Gao L Liu Z Chen L Zhang H. Effects of optimal Carbohydrase mixtures on nutrient digestibility and digestible energy of corn- and wheat-based diets in growing pigs. Animals. (2020) 10:1846. doi: 10.3390/ani10101846. PMID 33050555, PMID: 33050555 PMC7601035

[13] Graham H Hesselman K Aman P. The influence of wheat bran and sugar-beet pulp on the digestibility of dietary components in a cereal-based pig diet. J Nutr. (1986) 116:242–51. doi: 10.1093/jn/116.2.242, PMID: 3003295

[14] Ward NE. Debranching enzymes in corn/soybean meal-based poultry feeds: a review. Poult Sci. (2021) 100:765–75. doi: 10.1016/j.psj.2020.10.074, PMID: 33518131 PMC7858153

[15] Mirzaie S Zaghari M Aminzadeh S Shivazad M Mateos GG. Effects of wheat inclusion and xylanase supplementation of the diet on productive performance, nutrient retention, and endogenous intestinal enzyme activity of laying hens. Poult Sci. (2012) 91:413–25. doi: 10.3382/ps.2011-01686 22252355

[16] Baghban-Kanani P Hosseintabar-Ghasemabad B Azimi-Youvalari S Seidavi A Ayaşan T Laudadio V. Effect of different levels of sunflower meal and multi-enzyme complex on performance, biochemical parameters and antioxidant status of laying hens. S Afr J Anim Sci. (2018) 48:390–9. doi: 10.4314/sajas.v48i2.20

[17] Masey O'Neill HV Smith JA Bedford MR. Multicarbohydrase enzymes for non-ruminants. Asian Australas J Anim Sci. (2014) 27:290–301. doi: 10.5713/ajas.2013.13261, PMID: 25049954 PMC4093217

[18] Aviagen. Arbor acres parent stock handbook Aviagen Group. (2009).

[19] Benalywa ZA Ismail MM Shamsudin MN Yusop Z. Assessing the comparative advantage of broiler production in peninsular Malaysia using policy analysis matrix. Trop Anim Health Prod. (2019) 51:321–7. doi: 10.1007/s11250-018-1690-8, PMID: 30112733

[20] Gao F Jiang Y Zhou GH Han ZK. The effects of xylanase supplementation on performance, characteristics of the gastrointestinal tract, blood parameters and gut microflora in broilers fed on wheat-based diets. Anim Feed Sci Technol. (2008) 142:173–84. doi: 10.1016/j.anifeedsci.2007.07.008

[21] Biesek J Banaszak M Grabowicz M Wlazlak S Adamski M. Production efficiency and utility features of broiler ducks fed with feed thinned with wheat grain. Animals. (2022) 12:3427. doi: 10.3390/ani12233427. PMID 36496948, PMID: 36496948 PMC9738547

[22] Bennett CD Classen HL Riddell C. Feeding broiler chickens wheat and barley diets containing whole, ground and pelleted grain. Poult Sci. (2002) 81:995–1003. doi: 10.1093/ps/81.7.995, PMID: 12162361

[23] Pirgozliev VR Birch CL Rose SP Kettlewell PS Bedford MR. Chemical composition and the nutritive quality of different wheat cultivars for broiler chickens. Br Poult Sci. (2003) 44:464–75. doi: 10.1080/0007166031000085594 12964631

[24] Ma X Li Z Zhang Y. Effects of the partial substitution of corn with wheat or barley on the growth performance, blood antioxidant capacity, intestinal health and fecal microbial composition of growing pigs. Antioxidants. (2022) 11:1614. doi: 10.3390/antiox11081614. PMID 36009333, PMID: 36009333 PMC9405354

[25] Mavromichalis I Hancock JD Senne BW Gugle TL Kennedy GA Hines RH. Enzyme supplementation and particle size of wheat in diets for nursery and finishing pigs. J Anim Sci. (2000) 78:3086–95. doi: 10.2527/2000.78123086x 11132823

[26] Liu YF Zhao HB Liu XM You W Cheng HJ Wan FC. Substitution of wheat for corn in beef cattle diets: digestibility, digestive enzyme activities, serum metabolite contents and ruminal fermentation. Asian Australas J Anim Sci. (2016) 29:1424–31. doi: 10.5713/ajas.15.0866, PMID: 26954111 PMC5003967

[27] Noblet J Perez JM. Prediction of digestibility of nutrients and energy values of pig diets from chemical analysis. J Anim Sci. (1993) 71:3389–98. doi: 10.2527/1993.71123389x 8294292

[28] Stein H Pahm A Roth J. Feeding wheat to pigs. Swine Focus. (2010) 2:1–8.

[29] Hetland H Svihus B Olaisen V. Effect of feeding whole cereals on performance, starch digestibility and duodenal particle size distribution in broiler chickens. Br Poult Sci. (2002) 43:416–23. doi: 10.1080/00071660120103693 12195801

[30] Preston CM McKracken KJ McAllister A. Effect of diet form and enzyme supplementation on growth, efficiency and energy utilisation of wheat-based diets for broilers. Br Poult Sci. (2000) 41:324–31. doi: 10.1080/713654933, PMID: 11081428

[31] Parsaie S Shariatmadari F Zamiri MJ Khajeh K. Influence of wheat-based diets supplemented with xylanase, bile acid and antibiotics on performance, digestive tract measurements and gut morphology of broilers compared with a maize-based diet. Br Poult Sci. (2007) 48:594–600. doi: 10.1080/00071660701615788, PMID: 17952731

[32] Geyra A Uni Z Sklan D. The effect of fasting at different ages on growth and tissue dynamics in the small intestine of the young chick. Br J Nutr. (2001) 86:53–61. doi: 10.1079/BJN2001368, PMID: 11432765

[33] Yaghobfar A Kalantar M. Effect of non-starch polysaccharide (NSP) of wheat and barley supplemented with exogenous enzyme blend on growth performance, gut microbial, pancreatic enzyme activities, expression of glucose transporter (SGLT1) and mucin producer (MUC2) genes of broiler chickens. Rev Bras Ciên Avíc. (2017) 19:629–38. doi: 10.1590/1806-9061-2016-0441

[34] Barekatain R Chrystal PV Nowland T Moss AF Howarth GS Hao Van TT. Negative consequences of reduced protein diets supplemented with synthetic amino acids for performance, intestinal barrier function, and caecal microbiota composition of broiler chickens. Anim Nutr. (2023) 13:216–28. doi: 10.1016/j.aninu.2023.01.011, PMID: 37388459 PMC10300400

[35] Camilleri M Madsen K Spiller R Greenwood-Van Meerveld B Verne GN. Intestinal barrier function in health and gastrointestinal disease. Neurogastroenterol Motil. (2012) 24:503–12. doi: 10.1111/j.1365-2982.2012.01921.x 22583600 PMC5595063

[36] Hamaker BR Tuncil YE. A perspective on the complexity of dietary fiber structures and their potential effect on the gut microbiota. J Mol Biol. (2014) 426:3838–50. doi: 10.1016/j.jmb.2014.07.028, PMID: 25088686

[37] Ghazaghi M Hassanabadi A Mehri M. Apparent and standardized ileal amino acid digestibilities of corn, wheat, soybean meal, and corn gluten meal in quail chicks. Poult Sci. (2023) 102:102314. doi: 10.1016/j.psj.2022.102314, PMID: 36470030 PMC9719858

